# Smoking Prevalence and Associated Factors as well as Attitudes and Perceptions towards Tobacco Control in Northeast China

**DOI:** 10.3390/ijerph120708606

**Published:** 2015-07-22

**Authors:** Zhijun Li, Yan Yao, Weiqing Han, Yaqin Yu, Yawen Liu, Yuchun Tao, Changgui Kou, Lingling Jiang, Qing Sun, Yutian Yin, Huiping Zhang, Bo Li

**Affiliations:** 1 Department of Epidemiology and Biostatistics, Jilin University School of Public Health, Changchun Center for Disease Control and Prevention, Changchun 130000, China; E-Mails: beyond.hehe@163.com (Z.L.); yaoyan@jlu.edu.cn (Yan.Y.); hanwq1987@foxmail.com (W.H.); yuyaqin5540@163.com (Yaq.Y.); ywliu@jlu.edu.cn (Y.L.); taoyuchun@163.com (Y.T.); koucg@jlu.edu.cn (C.K.); jianglingling.2008@163.com (L.J.); Yinyt13@mails.jlu.edu.cn (Yut.Y.); 2 Department of Food and Nutrition, Jilin University School of Public Health, Changchun 130000, China; E-Mail: qingbao7@126.com; 3 Department of Psychiatry, Yale University School of Medicine, New Haven, CT 06511, USA; E-Mail: huiping.zhang@yale.edu

**Keywords:** smoking, environmental tobacco smoke, attitude, tobacco control

## Abstract

*Objectives*: The present study aimed to investigate the prevalence of smoking and environmental tobacco smoke (ETS), the associated factors of current smoking among adults, and their attitudes and perceptions towards tobacco control. *Methods*: A population-based cross-sectional survey was conducted in 2012 using a self-reported questionnaire. A representative sample of adults aged 18–79 years was collected in the Jilin Province of Northeast China by a multistage stratified random cluster sampling design. Descriptive data analysis was conducted, and 95% confidence intervals (CI) of prevalence/frequency were calculated to enable comparisons between the alleged differences and similarities. Multivariable logistic regressions were used to examine the risk factors associated with current smoking. *Results*: 21,435 adults responded to the survey (response rate: 84.9%). The overall prevalence of ever smoking, current smoking, and former smoking or smoking cessation was 39.1% (95% CI: 38.3–39.9), 31.8% (95% CI 31.1–32.6), and 7.3% (95% CI: 6.9–7.7), respectively. The proportion of ETS exposure among adult non-smokers in Jilin Province was 61.1% (95% CI: 60.1–62.1), and 23.1% (95% CI: 22.3–24.0) of the non-smokers reported daily ETS exposure. The proportion of ETS exposure at home was 33.4% (95% CI: 32.5–34.4), but the proportion of ETS exposure at restaurants was lower (6.5%) (95% CI: 6.0–7.1). More than 90% of the participants had positive attitudes and perceptions towards tobacco control, but 23.2% (95% CI: 22.5–24.0) of them did not agree with the perception of “smoking is fully quit in public places”, and almost half of the adults (49.5%) (95% CI: 48.7–50.3) did not agree with the perception of “hazards of low-tar cigarettes are equal to general cigarettes”. *Conclusions*: Smoking and exposure to ETS are prevalent among adults from the Jilin Province of Northeast China. Our findings suggest that tobacco control should be advocated in Northeast China. Anti-smoking campaigns and legislation should be built into the public health curriculum and government policy.

## 1. Introduction

Smoking is one of the major global public health problems. It is also the major preventable factor of smoking-related diseases, premature mortality, and general mortality [1,2,3]. Previous studies found that smoking was associated with cancers, cardiovascular diseases, respiratory system diseases, and other health problems [2,4]. At present, there are over one billion smokers worldwide, and the number of smokers is steadily increasing, especially in developing countries [5,6]. Smoking contributes to over five million deaths every year, and almost 50% of smokers die due to smoking-related diseases [7]. The World Health Organization [8] predicts that over eight million deaths will be attributable to smoking worldwide in 2030 [9], and about 10% of deaths of adults will be due to smoking-related diseases in European countries [10]. In addition, over one billion deaths may result from smoking at the end of this century if effective tobacco control measures are not taken [11]. China is the world’s largest tobacco producer and consumer. There are more than 300 million smokers, and one million deaths of smokers per year in China were caused by smoking-related diseases [12,13]. With the rapid social and economic development in past years, the prevalence of smoking and exposure to environmental tobacco smoke is high and steadily increasing in China, especially among females and adolescents. The 2010 China Global Adults Tobacco Survey showed that the smoking rate was 28.1% [12], and that of the 2002 national survey was 24.0% [14]. In 2002, 51.9% of non-smokers and, in 2010, 72.4% of non-smokers were exposed to ETS. 673,000 deaths in 2005 were due to smoking, and the number of smoking-related deaths is predicted to be over three million by the year 2050 [15]. Smoking and ETS exposure are prevalent in China and have become major contributors of smoking-related diseases and premature mortality.

Mounting evidence suggests that multiple risk factors are involved in smoking [16,17,18,19]. Previous studies indicate that smoking and ETS exposure are related to age, sex, socioeconomic status, and so on [20,21,22]. Age, education, and income are inversely correlated with smoking and ETS exposure [17,23]. Individuals with a low-level socioeconomic status are more likely to be smokers [24]. The attitudes and perceptions toward tobacco control may effectively influence smoking behaviors of adults [25,26], but there is little data to support the association between smoking and attitudes and smoking-related perceptions. 

The aim of our study was to investigate the prevalence of smoking and ETS exposure, the associated factors of current smoking among adults, and their attitudes and perceptions towards tobacco control in the Jilin Province of Northeast China.

## 2. Methods

A population-based cross-sectional survey was conducted among residents who were 18–79 years old and were living in Jilin Province for over six months. The multistage stratified cluster sampling method was used to select the study sample. Nine regions (Changchun, Jilin, Siping, Liaoyuan, Tonghua, Baishan, Songyuan, Baicheng, and Yanbian), 32 districts or counties, 95 towns or communities, and 45 units in Jilin Province were selected. The detailed stratifying process was reported previously [27]. Ethical approval was obtained by Jilin University School of Public Health, and written informed consent was obtained from all subjects.

The interviews were conducted by trained investigators. The questionnaire included information on demographics, smoking and ETS exposure status, and attitudes and perceptions towards tobacco control. “Current smokers” were defined as those who smoked any tobacco products during the investigation. “Former smokers” were defined as those who smoked in the past but have given up smoking for more than three months at the time of the investigation. “Ever smokers” included both current and former smokers. “ETS exposure subjects” were defined as those who perceived that they were exposed to or were exposed to second-hand smoke or a smoking environment from smokers during past seven days.

To make the sample representative of the population in Jilin Province, we used post-stratification adjustment according to the distribution of regional, urban/rural, age, and gender groups in the 2010 census of the adult population in Jilin Province. All statistical analyses were conducted based on the complex sampling design. Confidence intervals (CI) of 95% prevalence/frequency were calculated to enable comparisons between the alleged differences and similarities [28]. Rao-Scott Chi-square tests were used to compare the prevalence of smoking in different groups. In order to adjust for potential confounding effects, multiple regression analyses were carried out to find out independent factors associated with current smoking. Forward stepwise selection was used to select parameters with *p* < 0.05 to enter and *p* > 0.10 to exclude. The Cox and Snell test was used to evaluate the overall model performance, which performed steadily (Cox and Snell test: *p* = 0.265). In the final regression model, there 14 covariates were included to study the associations between socioeconomic characteristics and attitudes and perceptions towards tobacco control with current smoking among adults. All statistical analyses were performed using the complex sampling function of SPSS 22.0, and *p* ≤ 0.05 was considered to be statistically significant.

## 3. Results

For the study, 23,050 subjects aged over 18 years were recruited and 21,435 subjects completed the survey, resulting in a response rate of 84.9%. Response rates of urban and rural areas were 81.8% and 88.6%, respectively. The age of subjects ranged from 18 to 79 years (mean ± S.D.: 47 ± 13 years). The study subjects included 10,337 males (48.2%) and 11,098 females (51.8%). Of the subjects, 52.0% were from urban areas (11,152), 48.0% were from rural areas (10,283), and 92.7% were Han Chinese. The responses showed that 42.3% of the participants completed senior high school or a higher-level of education, 85.4% of the subjects were married or cohabitated, 54.9% of them were labor workers, and 30.9% of them had a family monthly income of 1000~2000 Chinese Yuan. 

The prevalence of smoking and ETS exposure status among Jilin Province adults in 2012 is shown in Table 1. The overall prevalence of ever smoking, current smoking, and former smoking or smoking cessation was 39.1% (95% CI: 38.3–39.9), 31.8% (95% CI: 31.1–32.6), and 7.3% (95% CI 6.9–7.7), respectively. The prevalence of smoking among males (64.8%) was significantly higher than that of females (11.7%) (*p* < 0.05). The overall prevalence of ETS exposure among adult non-smokers in Jilin Province was 61.1% (95% CI: 60.1–62.1), and it was 62.1% (95% CI: 60.5–63.7) for males and 60.6% (95% CI 59.3–61.8) for females. During the seven days prior to the investigation, 23.1% (95% CI: 22.3–24.0) of the non-smokers reported daily ETS exposure, 15.3% (95% CI 14.6–16.0) of them reported ETS exposure that lasted more than three days per week, and 22.2% (95% CI 21.3–23.0) of them reported ETS exposure that lasted less than three days per week. The proportion of ETS exposure was 33.4% (95% CI: 32.5–34.4) at home, 21.7% (95% CI: 20.8–22.5) at workplaces, and 11.1% (95% CI: 10.4–11.7) at entertainment places. However, the proportion of reported ETS exposure at restaurants was lower (6.5%) (95% CI: 6.0–7.1) than that of other places.

**Table 1:** Prevalence of smoking and environmental tobacco smoke (ETS) exposure among adults from Jilin Province, China in 2012.

	Male (n = 10,337) % (95% CI)[n]	Female (n = 11,098) % (95% CI)[n]	Total (n = 21,435) % (95% CI)[n]
Smoking status			
Ever	64.8 (63.6–65.9)[6779]	11.7 (11.1–12.4)[1664]	39.1 (38.3–39.9)[8443]
Current	52.9 (51.8–54.1)[5413]	9.4 (8.8–10.0)[1310]	31.8 (31.1–32.6)[6723]
Former	11.8 (11.2–12.5)[1366]	2.4 (2.1–2.7)[354]	7.3 (6.9–7.7)[1720]
ETS exposure frequency			
Everyday	17.9 (16.6–19.3)[816]	26.0 (24.9–27.1)[2560]	23.1 (22.3–24.0)[3376]
≥3 d/week	17.7 (16.4–19.1)[813]	13.9 (13.1–14.8)[1346]	15.3 (14.6–16.0)[2159]
1–3 d/week	25.5 (24.1–27.0)[1235]	20.3 (19.3–21.4)[1804]	22.2 (21.3–23.0)[3039]
Sources of ETS exposure			
Home	16.4 (15.2–17.7)[803]	42.9 (41.6–44.1)[4202]	33.4 (32.5–34.4)[5005]
Workplace	33.3 (31.8–35.0)[1496]	15.2 (14.3–16.2)[1305]	21.7 (20.8–22.5)[2801]
Restaurant	9.1 (8.2–10.1)[421]	5.1 (4.5–5.7)[417]	6.5 (6.0–7.1)[838]
Entertainment places	15.6 (14.4–16.8)[746]	8.6 (7.9–9.3)[760]	11.1 (10.4–11.7)[1506]
Other	3.6 (3.1–4.3)[176]	2.1 (1.8–2.5)[205]	2.7 (2.4–3.0)[381]

Note: Complex weighted computation was used in the statistical analysis.

Table 2 describes the prevalence of current smoking in relation to each survey item by gender. The overall current smoking prevalence among adults in Jilin Province was 31.8% (95% CI: 31.1–32.6), and the prevalence of current smoking among males (52.9%, 95% CI: 51.8–54.1) was significantly higher than that of females (9.4%, 95% CI: 8.8–10.0) (*p* < 0.05). The prevalence of current smoking in urban areas (30.7%, 95% CI: 29.7–31.8) was similar to that of rural areas (33.2%, 95% CI: 32.1–34.4). The age group of 50–59 years had the highest current smoking prevalence (34.3%, 95% CI: 32.9–35.7), and the current smoking prevalence in subjects with a lower level of education (33.6%, 95% CI: 32.1–35.1) was higher than that in subjects with a higher level of education (22.3%, 95% CI: 13.1–35.4). The separated/divorced subjects had the highest rate of current smoking for both males (73.2%, 95% CI 65.8–79.6) and females (19.0%, 95% CI 13.4–26.3). Manual labor workers had the highest prevalence of current smoking (males: 57.6%, 95% CI: 56.1–59.0; females: 12.2%, 95% CI: 11.3–13.2). The current smoking prevalence of females whose family monthly income was less than 500 Chinese Yuan was nearly five times higher than those with a monthly family income of 3000~4999 Chinese Yuan.

**Table 2:** Characteristics of current smoking status of adults from Jilin Province, China in 2012.

Characteristics	Male % (95% CI)[n]	Female % (95% CI)[n]	Total % (95% CI)[n]
Areas			
Urban	52.8 (51.3–54.4)[2910]	6.6 (5.9–7.4)[411]	30.7 (29.7–31.8)[3321]
Rural	53.0 (51.3–54.7)[2503]	12.7 (11.7–13.7)[899]	33.2 (32.1–34.4)[3402]
Ethnic			
Han	52.8 (51.6–54.0)[4996]	9.6 (9.0–10.3)[1245]	32.0 (31.2–32.8)[6241]
Mancu	52.3 (47.0–57.6)[272]	7.4 (5.6–9.8)[55]	29.5 (26.4–32.8)[327]
Mongolian	52.2 (34.9–69.0)[21]	3.3 (1.0–10.5)[3]	23.2 (14.4–35.1)[24]
Korean	64.5 (55.6–72.5)[90]	0.8 (0.1–5.3)[1]	35.7 (29.9–42.0)[91]
Hui	45.7 (32.1–60.0)[31]	7.3 (2.7–18.2)[6]	26.2 (18.3–35.8)[37]
Other	59.0 (15.2–92.0)[2]	—	28.7 (7.5–66.7)[3]
Age			
18–19	41.6 (32.6–51.1)[63]	3.6 (1.0–11.7)[3]	23.7 (18.2–30.2)[66]
20–29	56.8 (53.7–59.9)[776]	4.7 (3.3–6.7)[38]	32.5 (30.3–34.7)[814]
30–39	53.9 (51.3–56.5)[1009]	5.7 (4.7–7.0)[108]	31.8 (30.1–33.6)[1117]
40–49	57.7 (55.7–59.7)[1553]	9.2 (8.2–10.2)[341]	33.1 (31.8–34.4)[1894]
50–59	53.2 (51.1–55.4)[1272]	13.9 (12.6–15.2)[434]	34.3 (32.9–35.7)[1706]
60–69	43.1 (39.5–46.8)[596]	16.1 (14.4–18.0)[313]	29.1 (26.9–31.5)[909]
70–79	29.4 (25.2–33.9)[144]	16.6 (12.5–21.8)[73]	23.1 (20.1–26.4)[217]
Education			
Primary school and below	54.4 (52.0–56.9)[1180]	19.0 (17.7–20.4)[870]	33.5 (32.0–34.9)[2050]
Junior middle school	56.1 (54.1–58.2)[1710]	8.1 (7.0–9.4)[280]	33.6 (32.1–35.1)[1990]
Senior middle school	54.6 (52.5–56.7)[1640]	5.2 (4.2–6.3)[132]	33.2 (31.7–34.7)[1772]
College	44.8 (42.2–47.5)[868]	1.7 (1.1–2.7)[28]	25.6 (23.9–27.3)[896]
Under graduate and above	39.8 (24.3–57.6)[15]	—	22.3 (13.1–35.4)[15]
Marriage			
Married	52.5 (51.3–53.7)[4526]	9.1 (8.5–9.8)[1101]	31.3 (30.5–32.1)[5627]
Single	53.2 (49.8–56.6)[630]	5.5 (3.6–8.2)[30]	34.4 (31.8–37.1)[660]
Separated/Divorced	73.2 (65.8–79.6)[143]	19.0 (13.4–26.3)[33]	48.7 (43.2–54.1)[176]
Widowed	45.7 (39.2–52.4)[114]	17.8 (15.1–20.9)[146]	24.9 (22.1–27.9)[260]
Occupation			
Manual	57.6 (56.1–59.0)[6436]	12.2 (11.3–13.2)[5610]	38.4 (37.3–39.4)[12,046]
Intelligence	48.6 (46.2–51.1)[2167]	2.7 (2.0–3.6)[2044]	26.3 (24.8–27.9)[4211]
Retired	41.4 (38.3–44.6)[1734]	9.7 (8.6–11.0)[3444]	21.6 (20.0-23.2)[5178]
Family per capita income			
<500	51.6 (48.9–54.3)[901]	17.2 (15.7–18.7)[527]	32.6 (31.0–34.2)[1428]
500–	52.3 (49.6–55.0)[907]	11.2 (9.9–12.7)[307]	30.4 (28.8–32.1)[1214]
1000–	53.3 (51.1–55.4)[1683]	7.3 (6.3–8.4)[284]	30.5 (29.1–32.0)[1967]
2000–	55.6 (53.0–58.2)[1090]	5.0 (3.8–6.4)[82]	34.7 (32.8–36.6)[1172]
3000–	51.0 (47.5–54.6)[493]	3.5 (2.3–5.3)[30]	32.6 (30.0–35.4)[523]
5000–	51.6 (44.9–58.2)[151]	4.6 (2.2–9.4)[10]	33.5 (28.9–38.4)[161]
Unknown	48.6 (42.5–54.7)[188]	12.2 (8.8–16.6)[70]	29.2 (25.4–33.3)[258]
Total	52.9 (51.8–54.1)[5413]	9.4 (8.8–10.0)[1310]	31.8 (31.1–32.6)[6723]

Note: Complex weighted computation was used in the statistical analysis.

Table 3 summarizes the modes of adults’ attitudes and smoking-related perceptions. More than 90% of participants supported or agreed with the statement: *smoking should be banned in public places and restaurants*; *government should increase tobacco control efforts*; and *every cigarette smoked is harmful to health*. Overall, 88.2% (95% CI 87.7–88.7) and 76.8% (95% CI 76.0–77.5) of respondents agreed with the statements *tobacco is addictive* and *smoking is fully quit in public places*. Only 35.2% (95% CI: 34.4–36.0) of the adults agreed with the statement *hazards of low-tar cigarettes are equal to general cigarettes*. However, 23.2% (95% CI: 22.5–24.0) of the adults did not agree with *smoking is fully quit in public places*, and almost half of the adults (49.5%, 95% CI: 48.7–50.3) did not agree with *hazards of low-tar cigarettes are equal to general cigarettes*. There were no significant differences between male and female adults in the attitudes and perceptions towards tobacco control (*p* > 0.05).

**Table 3:** Attitudes and perceptions towards tobacco control among adults from Jilin Province, China in 2012.

	Male (n = 10,337) % (95% CI)[n]	Female (n = 11,098) % (95% CI)[n]	Total (n = 21,435) % (95% CI)[n]
**Attitudes**			
Smoking should be banned in public places	93.0 (92.3–93.6)[9660]	93.8 (93.2–94.4)[10,405]	93.4 (92.9–93.8)[20,065]
Government should increased tobacco control efforts	92.5 (91.8–93.1)[9640]	95.4 (94.8–95.9)[10,568]	93.9 (93.5–94.3)[20,208]
Smoking should be banned in restaurants	89.6 (88.8–90.3)[9381]	94.6 (94.0–95.2)[10,524]	92.0 (91.5–92.5)[19,905]
**Perceptions**			
Smoking is fully quit in public places	80.5 (79.6–81.4)[8385]	72.8 (71.7–73.8)[8130]	76.8 (76.0–77.5)[16,515]
Hazards of low-tar cigarettes are equal to general cigarettes	40.1 (39.0–41.3)[4102]	29.9 (28.8–31.0)[3251]	35.2 (34.4–36.0)[7,353]
Every cigarette smoked is harmful to health	95.6 (95.1–96.1)[9896]	94.9 (94.3–95.4)[10,529]	95.2 (94.9–95.6)[20,425]
Tobacco is addictive	88.5 (87.8–89.2)[9118]	87.8 (87.1–88.5)[9656]	88.2 (87.7–88.7)[18,774]

Note: Complex weighted computation was used in the statistical analysis.

As shown in Table 4, current smoking, gender, marriage, occupation, and education are significantly associated with the perception that *government should increase tobacco control efforts* by multivariable logistic regression. Male participants were more likely to smoke than females (OR/95% CI: 11.53/10.49–12.67), and single, separated or divorced, or widowed respondents were more likely to smoke than those who got married (OR/95% CI: 1.15/0.94–1.40 (single); 2.58/1.95–3.41 (separated or divorced); 1.59/1.27–2.02 (widowed)). Compared with labor workers, intelligent and retired people were less likely to smoke (OR/95% CI: 0.81/0.71–0.92 (intelligent); 0.68/0.60–0.77 (retired)). Respondents with a lower level of education were more likely to smoke than those with a higher level of education, and the risk of smoking was reduced when education levels were elevated. Respondents who did not agree that the *government should increase tobacco control efforts* were more likely to smoke than those who agreed with the statement. Respondents aged 45–54 years were more likely to smoke than those aged 18–24 years (OR/95% CI: 1.27/1.02–1.58), and the smoking risk of the 65–79 age group was reduced (OR/95% CI: 0.73/0.54–0.97). Respondents who did not agree with the perceptions or attitudes towards tobacco control were more likely to be smokers.

**Table 4:** Multivariate analysis of risk factors associated with current smoking among adults from Jilin Province, China in 2012.

Characteristic	*p*	OR	95% CI
Gender			
Female		1.000	
Male	**<0.001**	11.528	10.489–12.670
Age			
18~		1.000	
25~	0.641	1.052	0.852–1.299
35~	0.137	1.181	0.949–1.470
45~	**0.033**	1.269	1.020–1.580
55~	0.992	0.999	0.795–1.254
65~79	**0.028**	0.726	0.545–0.966
Areas			
Rural		1.000	
Urban	0.062	1.094	0.996–1.203
Marriage			
Married		1.000	
Single	**0.016**	1.149	0.945–1.398
Separated/ Divorced	**<0.001**	2.582	1.953–3.413
Widowed	**<0.001**	1.598	1.265–2.019
Occupation			
Manual		1.000	
Intelligence	**0.001**	0.807	0.713–0.915
Retired	**<0.001**	0.684	0.605–0.772
Education			
Primary school and below		1.000	
Junior middle school	**<0.001**	0.609	0.536–0.691
Senior middle school	**<0.001**	0.502	0.438–0.576
Under graduate and above	**<0.001**	0.316	0.264–0.378
Family per capita income			
＜500		1.000	
500~	**0.006**	0.829	0.726–0.948
1000~	0.138	0.906	0.795–1.032
2000~	0.859	1.013	0.874–1.175
3000~	0.067	0.847	0.710–1.011
5000~	0.363	0.882	0.674–1.155
Smoking should be banned in public places			
Agree		1.000	
Not sure	**<0.001**	0.517	0.377–0.709
Disagree	**0.023**	0.726	0.550–0.957
Uncertainty	0.640	1.143	0.653–2.003
Government should increase tobacco control efforts			
Agree		1.000	
Not sure	**<0.001**	1.979	1.516–2.582
Disagree	**0.001**	1.788	1.257–2.545
Uncertainty	**0.002**	2.088	1.314–3.316
Smoking should be banned in restaurants			
Agree		1.000	
Not sure	**<0.001**	2.428	1.847–3.193
Disagree	**<0.001**	4.147	3.199–5.377
Uncertainty	0.264	1.328	0.807–2.186
Smoking is fully quit in public places			
Known		1.000	
Unknown	**<0.001**	0.698	0.627–0.778
Hazards of low-tar cigarettes are equal to general cigarettes			
Correct		1.000	
Incorrect	**<0.001**	0.688	0.608–0.778
Unknown	**<0.001**	0.617	0.561–0.678
Every cigarette smoked is harmful to health			
Correct		1.000	
Incorrect	0.915	0.984	0.734–1.320
Unknown	0.848	1.028	0.773–1.367
Tobacco is addictive			
Correct		1.000	
Incorrect	0.123	1.142	0.965–1.351
Unknown	**<0.001**	0.408	0.328–0.509

## 4. Discussion

To our knowledge, this was the first large population-based survey to investigate the prevalence of smoking and associated factors in the Jilin Province of Northeast China. We found that the prevalence of smoking and ETS exposure for Jilin Province adults both at home and in public places was high. The ever and current smoking rates in Jilin Province were higher than the rates from the China Global Adults Tobacco Survey in 2010 (GATS, 28.1%) [12], which reported that 52.9% of males and 2.4% of females were current smokers, but lower than that reported by others [29,30]. Our study showed that the prevalence of former smoking among adults in Jilin Province was lower than that reported in 2010 GATS China (16.9%) and other cities [31,32]. The prevalence of ETS exposure among non-smokers was higher, especially at home and in workplaces, but lower than the rates reported by 2010 GATS China (72.4%, 67.3%, and 63.3%). The results were consistent with those reported in published studies [33]. However, exposure of ETS mostly occurred in women and children [34,35], suggesting an urgent need to develop tobacco control strategies of these populations.

More male smokers than females have been estimated worldwide, which is inconsistent with our study. The prevalence of current smoking among males was consistent with the rate observed in 2010 GATS China [12], but the current smoking rate in females was higher than the results in 2010 GATS China. Smoking is prevalent in both urban and rural populations in China, and males in rural areas are more likely to be smokers than males in urban areas. This may be due to the migration of “peasant-workers” from rural areas to urban areas. They may lack knowledge or perceptions of smoking hazards due to health and tobacco advertisements. Ethnic minorities have a similar risk of smoking as Han Chinese. This may be due to genetic heritability and shared environments, thus needing further investigation [36]. We found a lower prevalence of smoking among those aged 60 years and above, which is consistent with reports from other Asian countries [37,38,39]. This may be due to the reason that older people have less pressure and more time to accept health information and medical advice and confront smoking-related diseases, thus increasing the health consciousness following physical decline with age. Separated or divorced adults were more likely to be smokers than married ones. This finding is consistent with those reported by others [20,40]. This can be explained by marriage protection theories because married people have greater economic, social, and psychological support, while separated or divorced people have emotional distress that may lead them to become smokers for comfort. In this study, we found a significant association between profession and current smoking. Labor workers had a greater risk of smoking than those who are intelligent or retired people, and this finding was also reported in European and Asian populations [29,41,42]. A possible explanation is that labor workers have a lower socioeconomic status, more physical pressure, and psychosocial and emotional problems. Education levels and family per capita income were significantly associated with the prevalence of current smoking. People who had high levels of education and income were less likely to be smokers. This finding is similar to that reported previously [43]. Labor workers with a low level of education and income had a lower level of socioeconomic status. This group of people had financial stress and unhealthy lifestyles, and they lacked health care. 

In the study, we observed that most adults showed positive attitudes and perceptions towards tobacco control, but some perceptions towards tobacco control were still unclear among adults in the Jilin Province of Northeast China. Adults who support the statements *government should increase tobacco control efforts* and *smoking should be banned in restaurants* are protected from current smoking. However, people who do not agree with smoking-related perceptions appear to have current smoking behaviors. Therefore, the government should enhance tobacco control and take effective measures to change smoking attitudes and promote smoke-free legislation. The government should play the leading role in tobacco control programs. So far, China does not yet have a national smoke-free law; the new Beijing law is the strongest subnational tobacco control regulation adopted in the country to date. Since 1 June 2015, Beijing’s new smoke-free law has taken effect. Although Jilin Province does not implement similar control measures in public places, it also has local legislation to control smoking which took effect in 1996. Besides, measures, such as pictorial health warning, and educational campaigns, have been taken to control smoking in public places in Jilin.

Our study is the first large population-based survey on the prevalence of smoking and its associated risk factors in the Jilin Province of Northeast China. However, the present study has limitations. First, the status of smoking and attitudes or perceptions towards tobacco control were based on self-reporting, which may generate recall biases. Second, we did not make quantitative classifications in relation to attitudes and perceptions. Third, those who were too weak or ill to complete the interview were excluded. Fourth, since the participants were from Jilin Province, our findings cannot be extended to predict the prevalence of smoking in other regions of China.

## 5. Conclusions

The prevalence of smoking and exposure to ETS is higher than that reported in 2010 GATS China, and smoking and exposure to ETS are prevalent among adults in the Jilin Province of Northeast China. Most adults have positive attitudes and perceptions towards tobacco control, but some perceptions are still unclear. The results from the present study suggest an urgent need for tobacco control and an awareness of smoking-related hazards in the Jilin Province of Northeast China, and our findings could help the government develop effective tobacco control policies and promote smoke-free legislation.
