# Erratum: Li, Z., *et al*. Smoking Prevalence and Associated Factors as well as Attitudes and Perceptions towards Tobacco Control in Northeast China. *Int. J. Environ. Res. Public Health* 2015, *12*, 8606–8618

**DOI:** 10.3390/ijerph13030312

**Published:** 2016-03-11

**Authors:** International Journal of Environmental Research and Public Health Editorial Office

**Affiliations:** MDPI, Klybeckstrasse 64, Basel CH-4057, Switzerland; ijerph@mdpi.com; Tel.: +41-61-683-77-34

We wish to make the following correction to the published paper [1]. In the affiliation, the postcode and telephone number are incorrect. The correct affiliations are:

^1^ Department of Epidemiology and Biostatistics, Jilin University School of Public Health, Changchun 130021, China; beyond.hehe@163.com (Z.L.); yaoyan@jlu.edu.cn (Yan.Y.); hanwq1987@foxmail.com (W.H.); yuyaqin5540@163.com (Yaq.Y.); ywliu@jlu.edu.cn (Y.L.); taoyuchun@163.com (Y.T.); koucg@jlu.edu.cn (C.K.); jianglingling.2008@163.com (L.J.); Yinyt13@mails.jlu.edu.cn (Yut.Y.)

^2^ Department of Food and Nutrition, Jilin University School of Public Health, Changchun 130021, China; qingbao7@126.com

^3^ Department of Psychiatry, Yale University School of Medicine, New Haven, CT 06511, USA; huiping.zhang@yale.edu

* Correspondence: li_bo@jlu.edu.cn; Tel./Fax: +86-431-8561-9451

We apologize to the authors of the article and readers of the journal for any inconvenience.
